# BKCa channel inhibitor modulates the tumorigenic ability of hormone-independent breast cancer cells via the Wnt pathway

**DOI:** 10.3892/or.2014.3617

**Published:** 2014-11-24

**Authors:** BRANDON M. SCHICKLING, SARAH K. ENGLAND, NUKHET AYKIN-BURNS, LYSE A. NORIAN, KIMBERLY K. LESLIE, VICTORIA P. FRIEDEN-KOROVKINA

**Affiliations:** 1 Department of Obstetrics and Gynecology, University of Iowa, Iowa City, IA, USA; 2 Department of Urology, University of Iowa, Iowa City, IA, USA; 3 The Holden Comprehensive Cancer Center, University of Iowa, Iowa City, IA, USA; 4 Division of Basic Science Research, Washington University, St. Louis, MO, USA; 5 Division of Radiation Health, University of Arkansas for Medical Sciences, Little Rock, AR, USA

**Keywords:** BKCa channels, breast cancer, β-catenin, iberiotoxin

## Abstract

In breast cancers, the large conductance Ca^2+^ and voltage sensitive K^+^ (BKCa) channels have been hypothesized to function as oncoproteins, yet it remains unclear how inhibition of channel activity impacts oncogenesis. We demonstrated herein that iberiotoxin (IbTX), an inhibitor of BKCa channels, differentially modulated the *in vitro* tumorigenic activities of hormone-independent breast cancer cells. Specifically, in HER-2/neu-overexpressing UACC893 cells and triple-negative MDA-MB-231 cells, IbTX selectively attenuated anchorage-independent growth with concomitant downregulation of β-catenin as well as total and phosphorylated Akt and HER-2/neu. By contrast, HER-2/neu-overexpressing SK-BR-3 cells were insensitive to IbTX. Molecular analyses showed an absence of β-catenin and a dose-dependent upregulation of total and phosphorylated Akt and HER-2/neu in these cells. Taken together, these studies identify β-catenin as a putative modulator of the inhibitory actions of IbTX in sensitive breast cancer cells.

## Introduction

The large conductance calcium and voltage activated potassium (BKCa) channels have been shown to function as oncogenes in certain cancers (1–4). BKCa channels generate vast amounts of outward K^+^ currents and therefore are powerful modulators of the transmembrane potential of a cell. BKCa channels are overexpressed in many types of cancers via gene amplification, alternative splicing or increased protein half-life (5–8). In addition, neoplastic BKCa channels may possess augmented sensitivity to Ca^2+^ and voltage and hence generate K^+^ currents in environments where their normal counterparts are silent (5). The enhanced activity of BKCa channels shifts cellular transmembrane potential to favor proliferative phenotypes (9). It is therefore plausible for BKCa channels to be considered putative targets for anticancer therapies. The contributions of BKCa channels to cancer cell migration and invasion have been previously demonstrated; however, their role in tumorigenesis has not been investigated (1,3,10).

The present study presents novel findings that an inhibitor of BKCa channels, iberiotoxin (IbTX), selectively decreased anchorage-independent growth and tumorigenicity in breast cancer cells expressing β-catenin. Our data suggest that the attenuated tumorigenicity is a result of depolarizing shifts in cell transmembrane potential and subsequent downregulation of β-catenin and (phospho)Akt and HER-2/neu protein levels.

## Materials and methods

### Cell culture

UACC893, MDA-MB-231, SK-BR-3 and MCF10A cells were purchased from ATCC (Manassas, VA, USA). Cells were propagated in DMEM:F12 medium (Sigma-Aldrich, St. Louis, MO, USA) supplemented with 20% fetal bovine serum (FBS).

### Membrane potential assays

Cells were pre-loaded for 30 min with 2 μmol/l of a membrane potential-sensitive dye DiBAC4(3) (Invitrogen, Life Technologies, Carlsbad, CA, USA) in buffer containing (mmol/l): 20 HEPES, 140 NaCl, 2 KCl, 1 MgCl_2_, 2 CaCl_2_, 10 glucose prior to seeding onto mouse laminin (Sigma-Aldrich)-coated glass bottom 35-mm tissue culture dishes (MatTek Corporation, Ashland, MA, USA). After 24 h, dishes were placed on a microscope stage pre-heated to 37°C, and 10 nmol/l IbTX (Sigma-Aldrich) was added. Immediately following IbTX, cells were observed every minute for 20 min using the 488 nm laser of an LSM 510 Zeiss confocal scanning microscope (Zeiss, Jena, Germany). Signals from 5 to 7 regions of interest were quantified and averaged using LSM 510 image browser (Zeiss). Data were fitted using non-linear regression analysis and significant differences estimated by ANOVA and post hoc Bonferroni’s t-test (SigmaPlot; Systat Software, Inc., San Jose, CA, USA). A P-value ≤0.05 was considered to indicate a statistically significant result.

### Cell viability assays

Cells were seeded into 6-well cluster dishes at 100,000 cells/well in DMEM:F12 supplemented with 20% FBS in the absence or presence of IbTX (2, 5, 10, 25 and 50 nmol/l) for 48 h. Subsequently, cells were stained with trypan blue and counted using Countess Automated Cell Counter (Invitrogen). Experiments were repeated three times, and statistical differences were assessed using the Student’s t-test.

### Soft agar assays

Soft agar assays were performed as previously described (17). Briefly, following IbTX incubations as described for cell viability assays, cells were re-suspended in 0.3% agar in DMEM:F12 tissue culture medium supplemented with 20% FBS. Cell suspensions were seeded onto 0.5% basal agar in 6-well cluster dishes at 1,000 cells/well. The cell colonies were visualized 21 days later using crystal violet solution (crystal violet 0.005% and citric acid 0.1%) and manually counted. Experiments were repeated at least twice in quadruplicate.

### Western immunoblotting

Whole cell lysates were isolated using RIPA buffer containing (mmol/l) 150 NaCl, 50 Tris pH 7.4, 1 EDTA, 1% NP-40, 0.5% sodium deoxycholate, 0.1% SDS and protease and phosphatase inhibitors. Twenty micrograms of protein was separated on 4–20% Tris-HCl gels (Bio-Rad, Hercules, CA, USA), transferred on nitrocellulose membranes, and blocked in 5% BSA for 1 h at room temperature. Blots were then incubated with the rabbit anti-HER-2/neu antibody (1:500 EMD; Millipore, Billerica, MA, USA), rabbit β-catenin antibody (1:500), rabbit T308 phospho-Akt or total Akt antibody (both from Cell Signaling Technology, Danvers, MA, USA) overnight at 4°C. Signals were detected with IRDye 680 secondary antibodies (1:5,000) for 1 h at room temperature and visualized using LI-COR Odyssey Imaging System (both from LI-COR, Lincoln, NE, USA). Blots were subsequently incubated with mouse GAPDH antibody (1:1,000; Sigma) and anti-mouse IRDye 800CW secondary antibody (1:5,000; LI-COR) to ensure equal protein loading. N=3.

### RNA isolation and qPCR

Total mRNA was isolated using the mirVana miRNA isolation kit (Ambion, Life Technologies, Carlbad, CA, USA). Following isolation, the NanoDrop M-1000 was used to determine the concentration and the quality of the total RNA. A reverse transcription was performed using the SuperScript III First-Strand Synthesis kit (Invitrogen, Life Technologies) with 700 ng of total RNA. The gene expression was then determined with a SYBR-Green PCR assay (Applied Biosystems, Life Technologies, Carlbad, CA, USA) and run on the Applied Biosystems Model 7900 Genetic Analyzer. The data were normalized to the endogenous control 18s rRNA and analyzed using the program SDS 2.1 (Applied Biosystems, Life Technologies). Comparison of gene expression between the breast cancer MDA-MB-231, SK-BR-3 and UACC893 cells and the normal mammary MCF10A cells were completed using the ΔΔCt method. Primer sequences are available upon request.

### Fluorescent immunocytochemistry

Cells seeded onto mouse laminin-coated glass bottom 35-mm tissue culture dishes for 24 h were fixed in 2% paraformaldehyde for 30 min and permeabilized in 0.1% Triton X-100 for 4 min. Cells were blocked in phosphate-buffered saline (PBS) supplemented with 5% FBS + 1% NDS for 30 min at 37°C and incubated with the rabbit anti-BKCa antibody (Cell Signaling Technology) diluted 1:500 in 0.1% blocking buffer for 30 min at 37°C. The signal was detected using the donkey anti-rabbit DyLight 488 antibody (Jackson ImmunoResearch, West Grove, PA, USA) 1:1,000 in 0.1% blocking buffer for 15 min at 37°C. Cell nuclei were counterstained with propidium iodide (5 mg/ml 1:10,000) in PBS for 5 min at room temperature. Cells were visualized with a Zeiss 510 laser confocal microscope, and images were reconstructed using LSM Image Browser (both from Jena, Germany).

## Results

### BKCa channels contribute to resting transmembrane potential

**Figure 1:**
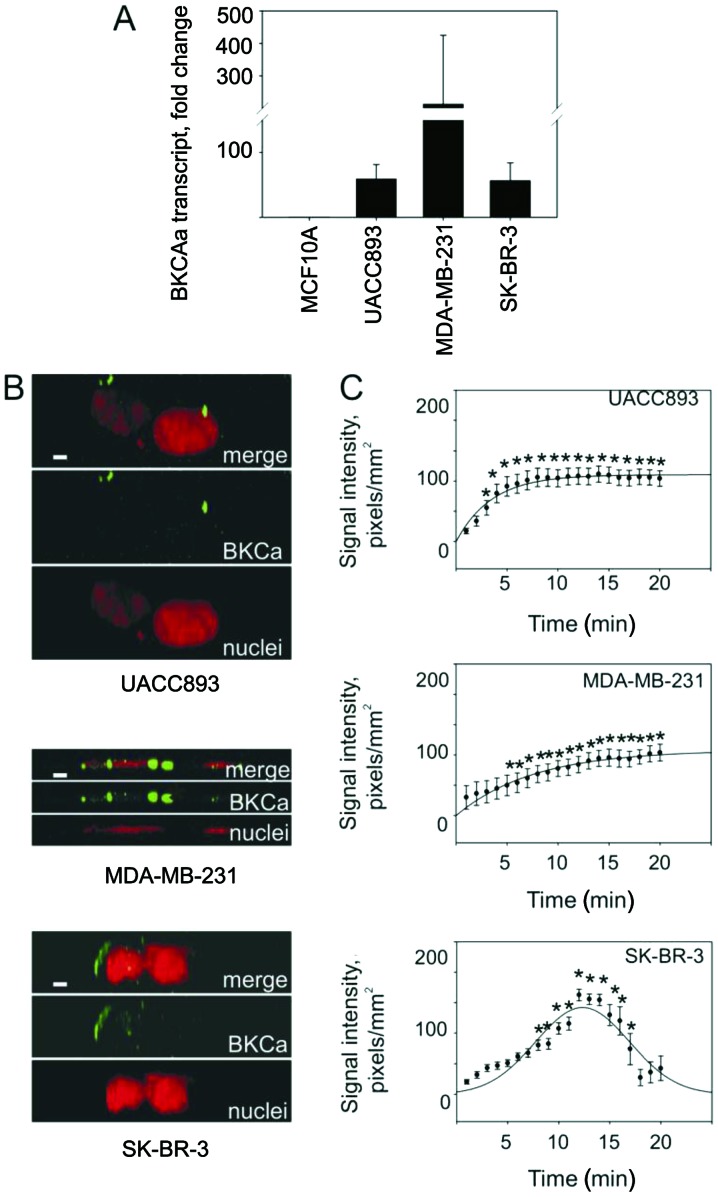
BKCa channels regulate the transmembrane potential in breast cancer cells. (A) Breast cancer cells displayed higher levels of BKCa transcripts compared to benign mammary epithelial cells. Total RNA from MCF10A benign mammary epithelial cells and UACC893, MDA-MB-231 and SK-BR-3 beast cancer cells was assayed for BKCa transcript levels using quantitative RT-PCR. Statistically significant differences vs. the MCF10A cells. N=3, P≤0.05. (B) BKCa channel proteins (green) were detected in UACC893 (upper panels), MDA-MB-231 (middle panels) and SK-BR-3 (lower panels) cells using fluorescent immunocytochemistry. Nuclei were counterstained with propidium iodide (red) and the image series were acquired using Zeiss 510 laser confocal microscope. A series was reconstructed to generate three dimensional images rotated to present the lateral sides of the cells with apical surfaces facing atop of their respective image. MDA-MB-231 cells did not possess basal-apical differentiation and appear flat in the images. Experiments were repeated at least three times for each cell line. (C) IbTX differentially modulates transmembrane potential. UACC893 (upper panel), MDA-MB-231 (middle panel) and SK-BR-3 (lower panel) cells pre-loaded with 2 μmol/l DiBac4(3) in membrane potential buffer and supplemented with 10 nmol/l IbTX were observed using a Zeiss 510 laser confocal microscope for 20 min with images captured every minute. Average signal intensities from 5 to 7 regions of interest were calculated and plotted. Increased signal intensity signifies depolarization due to IbTX inhibition of BKCa channels by IbTX. Significant differences vs. start of experiment (1 min). N=3. IbTX, iberiotoxin.

We began by using RT-PCR assays to establish the levels of BKCa channel mRNA in several types of malignant and non-malignant cell lines. We detected elevated levels of BKCa channel mRNA in breast cancer cell lines (UACC893, SK-BR-3 and MDA-MB-231) compared to these levels in the non-neoplastic MCF10A cells (Fig. 1A). BKCa channel proteins, detected using fluorescent immunocytochemistry, formed typical clusters on plasma membranes in all breast cancer cell models (Fig. 1B). Observations of live cells loaded with a membrane potential sensitive dye revealed that the selective BKCa channel inhibitor IbTX elicited cellular depolarization indicative of a presence of functional channels. Indeed, IbTX depolarized cells in all three models albeit to a different magnitude (Fig. 1C). The UACC893 cells were significantly depolarized within 4 min after IbTX addition, after which depolarization plateaued for the duration of the experiment. The MDA-MB-231 cells did not acquire a plateau phase but rather steadily increased their transmembrane potential throughout the 20-min observation period. SK-BR-3 cells were depolarized only transiently. The emergence of depolarizing phases, despite differences in duration, suggests that the BKCa channels regulate the resting transmembrane potential of all three breast cancer cell lines examined.

### BKCa channel inhibition selectively modulates anchorage-independent growth

**Figure 2:**
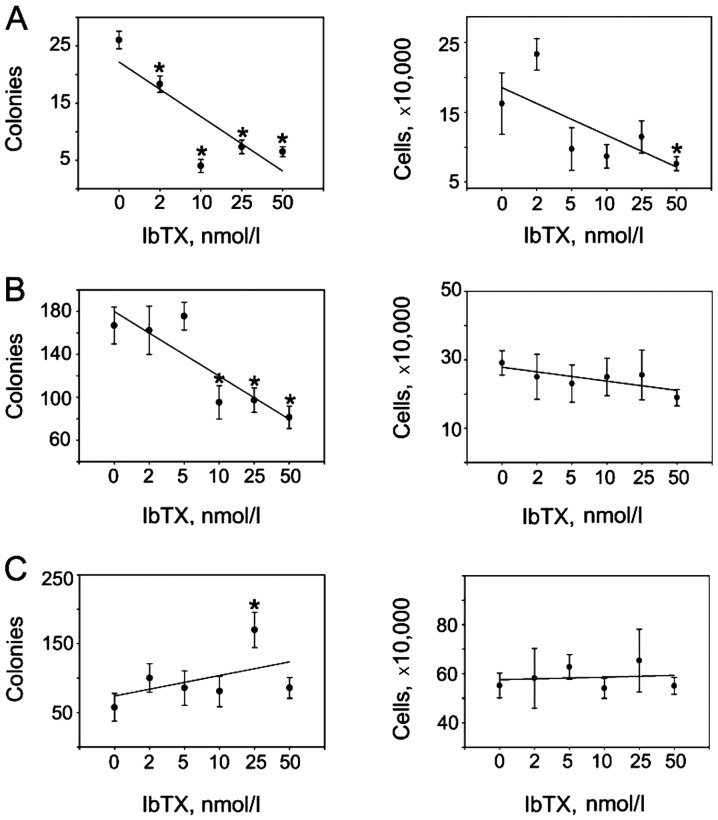
IbTX selectively modulates anchorage-independent clonogenic growth. Cells were incubated with increasing concentrations of IbTX for 48 h, stained with trypan blue and counted. Cells subsequently were seeded in agar-supplemented tissue culture media. Cell colonies were visualized with crystal violet staining and counted. (A) In UACC893 cells, IbTX inhibited anchorage-independent colony growth in soft agar (left panel). Significant attenuation of anchorage-dependent cell proliferation was observed only at 50 nmol/l (right panel). (B) IbTX suppressed clonogenic growth of MDA-MB-231 cells (left panel) without exhibiting inhibitory effects on cell proliferation (right panel). (C) IbTX did not modulate anchorage-dependent (left panel) or anchorage-independent (right panel) growth of SK-BR-3 cells. All assays were repeated at least twice in quadruplicate. Significant differences vs. IbTX-free controls (x-axis, 0). IbTX, iberiotoxin.

Given that BKCa channels were expressed and functional in our cancer cell lines, we asked whether pharmacological inhibition of these channels could lessen malignant phenotypes by impacting the ability of cells to proliferate, survive and/or form tumors *in vitro*. We found IbTX to attenuate *de novo* cell colony development in the UACC893 cells (Fig. 2A, left upper panel) with minimal inhibitory effects on anchorage-dependent cell proliferation (Fig. 2A, right upper panel). A similar dynamic was observed in the MDA-MB-231 cells where clonogenic growth (Fig. 2B, left middle panel) but not anchorage-dependent proliferation (Fig. 2B, right middle panel) was inhibited by IbTX. On the contrary, IbTX was ineffective in modulating colony formation (Fig. 2C, left lower panel) or proliferation (Fig. 2C, right lower panel) of SK-BR-3 cells. Thus, IbTX appeared to specifically reduce the tumorigenic features of the neoplastic phenotype in selected breast cancer models.

### BKCa channel inhibition modulates oncogenic pathways

**Figure 3:**
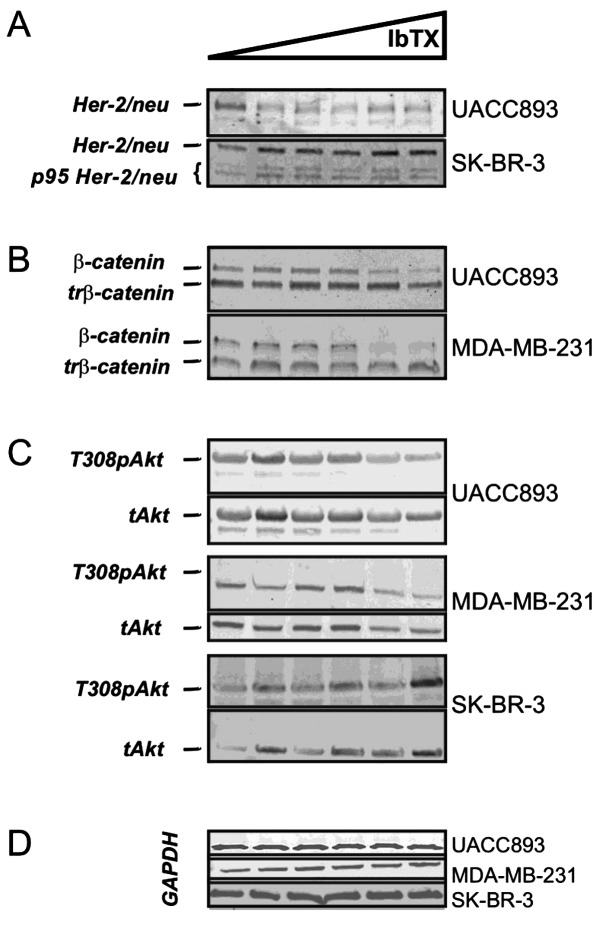
IbTX differentially regulates oncogenic pathways. (A) IbTX attenuated the HER-2/neu levels in the UACC893 cells (*HER-2/neu*, *UACC893*). SK-BR-3 cells express full length (*HER-2/neu*, *SK-BR-3*) and truncated (*p95 HER-2/neu*, *SK-BR-3*) oncoprotein isoforms with both being increased by IbTX. (B) IbTX downregulated the full length (*β-catenin*) but not truncated (*trβ-catenin*) β-catenin isoforms in the UACC893 and MDA-MB-231 cells. (C) In the UACC893 cells, IbTX attenuated phosphorylated (*T308pAkt*, *UACC893*) but not total Akt (*Akt*, *UACC893*). Both phosphorylated (*T308pAkt*, *MDA-MB-231*) and total (*Akt*, *MDA-MB-231*) Akt were decreased in the MDA-MB-231 cells with IbTX. In SK-BR-3 cells, phosphorylated (*T308pAkt*, *SK-BR-3*) and total (*Akt*, *SK-BR-3*) Akt increased at high IbTX concentrations. (D) GAPDH was used in the UACC893, MDA-MB-231 and SK-BR-3 cell immunoblotting to ensure equal protein loading. IbTX, iberiotoxin.

Both UACC893 and SK-BR-3 cells express the HER-2/neu gene. Thus, it was possible that disparate trends in their tumorigenic potential following IbTX treatment may have resulted from IbTX-induced modulations of oncoprotein levels. HER-2/neu levels decreased in the UACC893 cells at growth-inhibiting IbTX concentrations (Fig. 3A). By contrast, SK-BR-3 cells, which express both the long and short isoforms of HER-2/neu, demonstrated increased HER-2 levels after IbTX addition (Fig. 3A) (11). However, changes in HER-2/neu oncoprotein expression cannot account for the attenuated tumorigenic ability of triple-negative MDA-MB-231 cells, thus suggesting alternative mechanisms of growth regulation.

Upon further investigation, we found that cells with reduced tumorigenicity expressed β-catenin (Fig. 3B, UACC893 and MDA-MB-231). IbTX-resistant SK-BR-3 cells were β-catenin-negative (data not shown); these findings are consistent with earlier reports (12). In UACC893 and MDA-MB-231 cells, both long and truncated isoforms of β-catenin were identified (Fig. 3B, β-catenin and trβ-catenin), although IbTX decreased expression of only the long isoform (90 kDa) (13). The protein kinase Akt is known to phosphorylate β-catenin and regulate its activity (14). We therefore examined Akt levels in UACC893 and MDA-MB-231 cells treated with IbTX. In the UACC893 cells, T308 phospho-Akt was diminished with increasing concentrations of IbTX; in MDA-MB-231 cells both total and phospho-Akt were downregulated, concordant with β-catenin levels. In contrast, SK-BR-3 cells demonstrated fluctuating levels of phospho- and total-Akt that increased at higher IbTX concentrations (Fig. 3C). Hence, β-catenin-dependent pathway(s) may mediate the inhibitory actions of IbTX with regard to clonogenic potential in breast cancer cells.

## Discussion

BKCa channels have previously been proposed as targets for anticancer therapies in brain and prostate cancers due to their presumptive pro-oncogenic roles. However, their contributions to the pathogenesis of hormone-independent breast cancers are not well understood (1). The present study implies that BKCa channels may exert either pro- or anti-growth effects, depending on the particular molecular composition of the cancer cell type in question.

The growth-modulating roles of BKCa channels became apparent in our experiments when we explored anchorage-independent cell growth. These findings are in accord with earlier reports that found the BKCa channels modulated the tumorigenic potential of subpopulations of cancer cells (15). Molecular studies provided insights into the mechanisms used by BKCa channels to differentially regulate the tumorigenic properties of breast cancer cells. IbTX is a potent and selective BKCa channel inhibitor with minimal off-target effects at concentrations within the inhibitor’s selectivity range (16). In addition, cells with reduced tumorigenicity, i.e. UACC893 and MDA-MB-231, uniformly developed sustained transmembrane depolarization when IbTX was present. It is therefore plausible that suppression of oncogene(s) levels, and hence tumor formation, are secondary events occurring subsequent to sustained increases in transmembrane potential.

Notably, we also observed that BKCa channel downregulation via siRNAs did not recapitulate the inhibitory actions of IbTX on *in vitro* tumorigenesis despite satisfactory transfection levels (data not shown). These findings lend further credence to the importance of transmembrane potential for tumorigenesis as siRNAs are far less efficient in inhibiting K^+^ currents compared to IbTX (17). TEA, a non-selective blocker of K^+^ channels and depolarizing agent, has been shown to hamper *in vitro* endometrial tumorigenesis akin to IbTX in breast cancer models (18). A single discrepancy, namely the enhanced tumorigenic activity upon TEA washout that did not occur with IbTX, was due to the lower dissociation constant of IbTX compared to TEA, thus ensuring IbTX retention in soft agar (18). Hence, inhibitors with dissimilar chemical and pharmacological profiles that share similar depolarizing actions can equivalently modulate anchorage-independent growth in disparate cancer models, suggesting that transmembrane potential mediates their inhibitory effects.

In the present study, tumor cell lines that showed suppressed *in vitro* colony growth in the presence of IbTX shared positivity for β-catenin. Thus, it is possible that a canonical Wnt pathway could mediate the anti-tumorigenic actions of IbTX. BKCa/β-catenin complexes have been reported in cells of neural origin and in overexpression systems where β-catenin determines BKCa channel surface expression and clustering (19). However, in our experiments, IbTX downregulated β-catenin levels without significantly affecting the expression patterns of the BKCa channels. The observed differences were not due to the presence of BKCa splice variants with alterations in the β-catenin binding S10 domain (19). Moreover, β-catenin-negative SK-BR-3 cells not only have BKCa channels present on the cell surface, but also respond to IbTX with β-catenin-independent increases in the expression of BKCa channels. These findings imply that breast cancer cells and neural cells/heterologous systems utilize disparate mechanisms to sustain BKCa channel expression. Canonical Wnt signaling may be a prerequisite for the antitumor effects of BKCa channel inhibitors; however, mechanisms other than BKCa/Wnt interactions may also mediate these effects. Similarly, differential regulation of HER-2/neu oncoprotein levels by IbTX cannot be ascribed solely to previously reported HER-2neu/β-catenin complexes at least in SK-BR-3 cells (20).

In conclusion, the present study suggests that: i) BKCa channels function as oncogenes in β-catenin-positive breast cancer cells; ii) they direct their oncogenic input towards sustaining the tumorigenic ability of cancer cells; and iii) inhibitors of BKCa channels may modulate *in vitro* tumorigenesis via transmembrane depolarization.
